# Structural, optical, and magnetic studies of manganese-doped zinc oxide hierarchical microspheres by self-assembly of nanoparticles

**DOI:** 10.1186/1556-276X-7-100

**Published:** 2012-02-02

**Authors:** Yao-Ming Hao, Shi-Yun Lou, Shao-Min Zhou, Rui-Jian Yuan, Gong-Yu Zhu, Ning Li

**Affiliations:** 1 Key Lab for Special Functional Materials of Ministry of Education, Henan University, Kaifeng, 475004, People's Republic of China

**Keywords:** Mn-doped ZnO, hierarchical microspheres, optical properties, magnetic properties

## Abstract

In this study, a series of manganese [Mn]-doped zinc oxide [ZnO] hierarchical microspheres [HMSs] are prepared by hydrothermal method only using zinc acetate and manganese acetate as precursors and ethylene glycol as solvent. X-ray diffraction indicates that all of the as-obtained samples including the highest Mn (7 mol%) in the crystal lattice of ZnO have a pure phase (hexagonal wurtzite structure). A broad Raman spectrum from as-synthesized doping samples ranges from 500 to 600 cm^-1^, revealing the successful doping of paramagnetic Mn^2+ ^ions in the host ZnO. Optical absorption analysis of the samples exhibits a blueshift in the absorption band edge with increasing dopant concentration, and corresponding photoluminescence spectra show that Mn doping suppresses both near-band edge UV emission and defect-related blue emission. In particular, magnetic measurements confirm robust room-temperature ferromagnetic behavior with a high Curie temperature exceeding 400 K, signifying that the as-formed Mn-doped ZnO HMSs will have immense potential in spintronic devices and spin-based electronic technologies.

## Introduction

Zinc oxide [ZnO] exhibits many fascinating properties [1-7]. It is an intrinsic *n*-type II-VI semiconductor with a wide bandgap of 3.37 eV at room temperature [RT] and a large excitation binding energy of 60 meV. Because of these properties, ZnO presents a strong excitonic UV light emission at RT [2-6]. It also presents a high photoconductivity and considerable piezoelectric and pyroelectric properties [1]. Because of these properties, ZnO has attracted much attention for potential applications in various electronic and optoelectronic devices. In particular, the interest in ZnO has significantly been increased in the last decade [7-21] since the theoretical prediction of above RT ferromagnetism [RTFM] in Mn-doped ZnO diluted magnetic semiconductors [DMSs] by Dietl et al. [8]. DMSs are materials that simultaneously exhibit ferromagnetic and semiconducting properties. In DMS materials, magnetic transition ions substitute a small percentage of cation sites of the host semiconductor and are coupled with free carriers to yield ferromagnetism via indirect interaction [9-12]. DMSs are considered to be very important materials in future semiconductor spintronic applications due to the simultaneous control of 'electron' charge and spin [9-12]. Among all the magnetic transition ion-doped ZnO systems, Mn doping is usually the single most concerned mainly because of the fact that the thermal solubility of metallic Mn is larger than 10 mol% in ZnO, and the 'electron effective mass' is as large as approximately 0.3 me, where 'me' is the free-electron mass [13]. Therefore, injected spins and carriers in the nanostructures can be large, thus making Mn-doped ZnO ideal for the fabrication of spintronic nanodevices. For practical applications, a high-performance DMS with a high Curie temperature [*T*_C_] and saturation magnetic moments [*M*_S_] is required. However, until now, the mechanism involved in ferromagnetism [FM] is complicated and the reproducibility of ferromagnetic behavior is still a challenging problem. Because several groups have obtained different properties such as paramagnetism, anti-FM, and FM in Mn-doped ZnO [12,13,19-21], these magnetic properties are strongly dependent on the sample preparation conditions. Therefore, the development of a more controllable and repeatable synthetic route for RTFM Mn-doped ZnO nano/microstructures is crucial to their practical applications.

The fabrication of hierarchical and self-assembly (self-aggregation) micro-/nanostructures using nanoparticles, nanorods, nanoplatelets, etc. as building blocks at different levels have become a hot topic in recent material research fields [14-16]. Self-assembly and/or self-aggregation are fundamental mechanisms by which different nanoparticle assembly motifs or even close-packed periodic structures form in materials through spatial arrangement of their fundamental building blocks. The forces that controlled the assembly are determined by competing noncovalent intramolecular or intraparticulate interactions. The hierarchical structures obtained through the assembly of nanocrystalline building blocks provide new opportunities for optimizing, tuning, and/or enhancing the properties and performance of the materials [14-16]. So far, considerable efforts have been devoted to synthesize Mn-doped ZnO systems with RTFM including nanoparticles, nanowires, and thin films using different methods such as pulsed laser deposition, magnetron co-sputtering, and chemical vapor deposition [17-19]. However, there are very few reports on the synthesis of hierarchical spherical superstructures of Mn-doped ZnO DMS materials in solution phase. Herein, we only use zinc acetate and manganese acetate as precursors and ethylene glycol [EG] as solvent to synthesize Mn-doped ZnO hierarchical microspheres [HMSs] by self-assembly of nanoparticles. In particular, magnetic measurements confirm robust RTFM behavior with a high *T*_C _over 400 K. To our knowledge, there is no report on the Mn-doped ZnO hierarchical spherical structures showing a robust RTFM behavior with a high *T*_C_.

Meanwhile, optical properties of Mn-doped ZnO are currently the subject of numerous investigations in response to a strong demand for nano-/microscale magneto-optic devices in the future. However, it is unfortunate that although most of the Zn/Mn bulk and nanostructure materials exhibit RTFM, strong UV photoluminescence [PL] is hardly achieved. This may be due to the difficulty in controlling the interaction between the Mn dopant and intrinsic defects such as oxygen vacancies during the fabrication process [22-26]. So far, its luminescence mechanism has still been in discussion [22-26]. With the aim of providing further understanding of the optical nature of Mn-doped ZnO HMSs, UV-visible [vis] and PL spectra are used to study their optical characteristics, and the corresponding mechanism has been discussed. Also, the surface morphology of the products was investigated by scanning/transmission electron microscopy [SEM/TEM] or by high-resolution TEM [HRTEM]. The structure was studied by X-ray diffraction [XRD] and using Raman and Fourier transform infrared spectroscopy [FTIR] spectra.

## Experimental section

Zinc acetate [Zn(CH_3_COO)_2_·2H_2_O], manganese acetate [Mn(CH_3_COO)_2_·2H_2_O], and EG are analytic grade reagents and purchased without further treatment. In a typical process, 4 mmol of mixed reactants of [Zn(CH_3_COO)_2_·2H_2_O] with different amounts of [Mn(CH_3_COO)_2_·2H_2_O] was dissolved in 30 mL of EG. The mixture was stirred vigorously for 1 h, sealed in a Teflon-lined stainless steel autoclave of 50-mL capacity kept at 180°C for 5 h, and then allowed to cool to RT naturally. Yellow Mn-doped ZnO precipitates were centrifugally collected and rinsed with absolute ethanol several times. Finally, the precipitates were dried in air at 60°C overnight.

The morphologies and microstructures of these as-fabricated specimens were investigated by SEM/TEM (JSM5600LV, JEOL 2010, JEOL Ltd., Akishima, Tokyo, Japan), selected area electron diffraction [SAED], and HRTEM (JEOL Ltd, Akishima, Tokyo, Japan). The sample phases, crystal structures, chemical compositions, and element valences of the Mn-doped ZnO samples were detected by XRD (Phillips X'Pert Pro MPD, PANalytical B.V., Almelo, The Netherlands), Raman spectroscopy (microscopic confocal Raman spectrometer, RW-1000, Renishaw, Wotton-under-Edge, UK), and X-ray photoelectric spectrum [XPS] (KRATOS AXIS ULTR, Kratos Analytical, Ltd., Manchester, UK), respectively. RTPL measurement was carried with a fluorescence spectrophotometer (SPEX F212, Spex Industries, Metuchen, NJ, USA) with an Xe lamp as the excitation light source (330 nm). UV-vis absorption was carried with a UV-vis absorption spectrometer (Lambda35, PerkinElmer, Boston, MA, USA). The molecular structure of the as-synthesized samples was studied using FTIR spectra (Shimadzu-8700, Shimadzu Corporation, Nakagyo-ku, Kyoto, Japan). Magnetic properties were carried out using Quantum Design's superconductor quantum interference device [SQUID] (MPMS XL7, San Diego, CA, USA).

## Results and discussion

**Figure 1:**
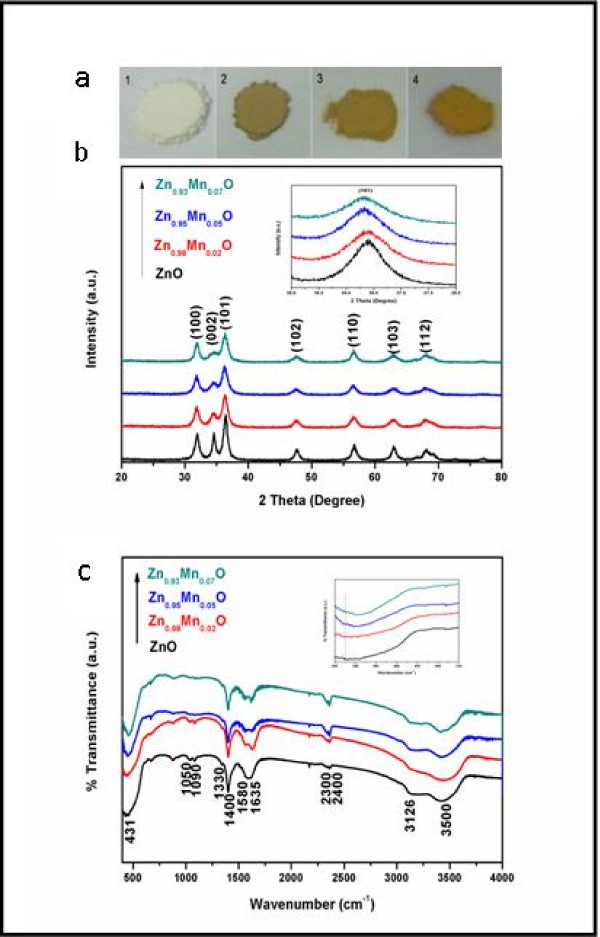
**Macrographs (a), XRD (b), and FTIR (c) spectra patterns**.

The macrographs of Zn_1-*x*_Mn*_x_*O HMSs (*x *= 0, 0.02, 0.05, and 0.07) are demonstrated in Figure 1a. We can find that the pure ZnO samples present a white color, while Mn-doped ZnO HMSs for different concentrations become light yellow, and the color becomes deeper and deeper with increasing Mn. The phase purity and crystal structure of the pristine ZnO and Mn-doped ZnO for various doping levels have been analyzed by XRD. All of the diffraction peaks can be indexed to the wurtzite structure of ZnO (space group P63mc; Figure 1b). The data are in agreement with the Joint Committee on Powder Diffraction Standards' card for ZnO (36-1451). There is no indication of any secondary phase or clusters, confirming that the samples are only one single phase. The XRD results also indicate that the Mn^2+ ^ions systematically substituted for the Zn^2+ ^ions in the sample without changing the wurtzite structure, as shown in the inset of Figure 1b. The most intense diffraction peak (101) is clearly evident with a slight shift into low angular scale, whereas the corresponding intensity decreases compared with undoped ZnO. Furthermore, the intensity of the diffraction peaks decreases, and the width broadens due to the formation of smaller grain diameters as a result of an increase in disorder on Mn^2+ ^doping. Also, the evolution of unit cell parameters as a function of Mn content has been given in Table 1. The overall trend shows that the lattice parameters are increasing with an increase of Mn content. This is consistent with the fact that the ionic radius of Mn^2+ ^is 0.80 Å, whereas that of Zn^2+ ^is 0.74 Å. The shifting and broadening of XRD lines with doping strongly suggest that Mn^2+ ^successfully substituted into the ZnO host structure at the Zn^2+ ^site.

**Table 1:** The unit cell parameters

Material	*a *(nm)	*c *(nm)
Pure ZnO	0.32425	0.52007
Zn_0.98_Mn_0.02_O	0.32663	0.52536
Zn_0.95_Mn_0.05_O	0.32914	0.53292
Zn_0.93_Mn_0.07_O	0.33208	0.53819

The composition, quality, and molecular structure of the product were analyzed by FTIR spectroscopy. FTIR measurements of all the samples were performed in the wave number range from 400 to 4,000 cm^-1 ^using the KBr method at RT as shown in Figure 1c. The broad absorption peak around 3,500 cm^-1 ^represents the stretching vibration of the O-H group. The absorption peaks observed between 2,300 and 2,400 cm^-1 ^are assigned to the CO_2 _mode [27]. The CO_2 _modes are present in the FTIR spectra not owing to the serious contamination in Mn-doped ZnO HMSs, but these modes may be due to atmospheric CO_2 _in the samples. Samples might have trapped some CO_2 _from the atmosphere during FTIR characterization which might have given such modes [27]. Two principal absorption peaks at 1,580 and 1,400 cm^-1 ^with a wave number separation of 180 cm^-1 ^correspond to the asymmetric stretching *υ*_as _(COO^-^) and symmetric stretching *υ*_s _(COO^-^) vibrations of unidentate acetate species [28]. These characteristic bands of 3,126, 1,090, and 1,050 cm^-1 ^are attributed to a neutrally adsorbed EG on the surface without an obvious shift in wave numbers compared with pure EG. The band at 1,330 cm^-1 ^is assigned to the stretching vibrations of *δ *(CH_3_) [28]. The bending vibration of the interlayer water molecule appeared with the typical band at 1,635 cm^-1^. The absorption band at 431 cm^-1 ^is assigned to the stretching mode of ZnO [29]. However, in the case of the Zn_1-*x*_Mn*_x_*O HMSs (*x *= 0.02, 0.05, and 0.07), the values of absorption bands are found to be blueshifted at 438, 445, and 456 cm^-1^, respectively. The enlarged spectrum in the wave number range is shown in the inset of Figure 1c. The change in the peak position of the ZnO absorption bands reflects that the Zn-O-Zn network is perturbed by the presence of Mn in its environment.

**Figure 2:**
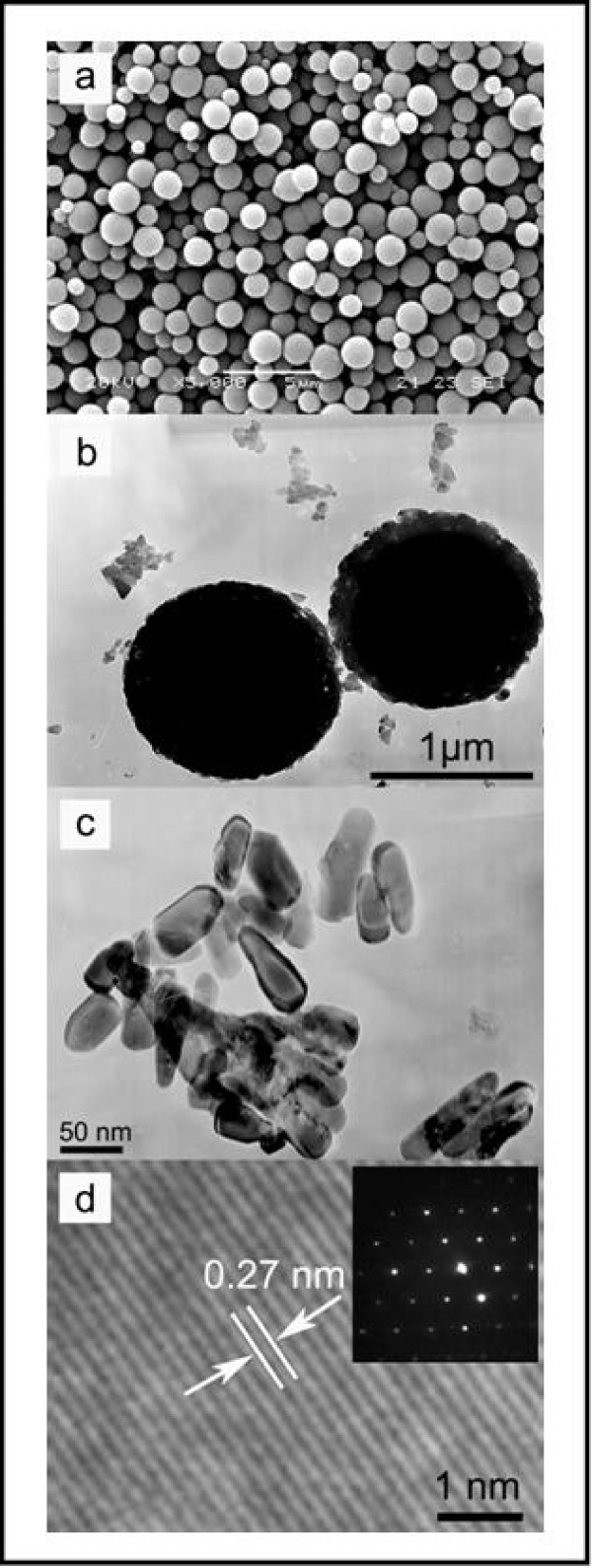
**SEM, TEM (a, b), HRTEM (c), and SAED (inset) (d) for Zn_0.93_Mn_0.07_O HMSs**.

The morphology and further structural characterization of as-obtained Mn-doped ZnO HMSs have been carried out using SEM/TEM. Large-scaled and monodispersed Zn_0.93_Mn_0.07_O HMSs observed in Figure 2a have been successfully prepared. The TEM images (Figure 2b) for a representative sample depict that the mean diameter of the Zn_0.93_Mn_0.07_O HMSs is about 1 to 1.5 μm. The high magnifying TEM micrograph (Figure 2c) clearly shows that the samples are composed of closely packed small nanoparticles. The lattice spacing ((002) for 0.27 nm) of Zn_0.93_Mn_0.07_O HMSs observed throughout the HRTEM image (Figure 2d) is a little larger than that ((002) for 0.26 nm) of the undoped ZnO HMSs (not shown), which is due to the bigger ionic radius of Mn (0.80 Å) than that of Zn (0.74 Å) and due to more structural defects. The absence of any impurity phase such as Mn and Mn-based secondary phases in the nanosize range has been further confirmed by SAED studies (inset of Figure 2d).

**Figure 3:**
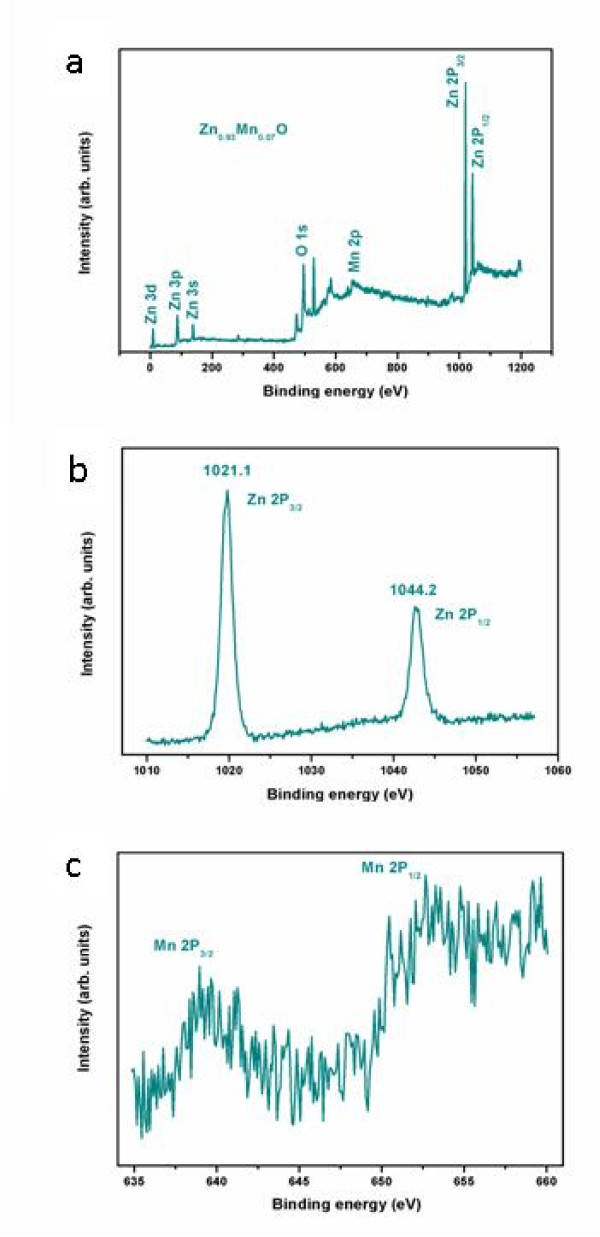
**XPS for Zn_0.93_Mn_0.07_O HMSs**. The elements of Zn, Mn, O, and adventitious C (**a**). The high-resolution XPS spectra of Zn (**b**) and Mn (**c**) species.

The XPS spectra were used to investigate the composition and chemical bond configuration of the Zn_0.93_Mn_0.07_O HMSs. The binding energies are calibrated by the carbon C_1s _peak (284.6 eV). The elements of Zn, Mn, O, and adventitious C can be detected as shown in Figure 3a. The high-resolution XPS spectra of Zn and Mn species are further illustrated in Figure 3b, c, respectively. In Figure 3b, two strong peaks that appeared at 1,044.2 and 1,021.1 eV can be attributed to Zn2p_3/2 _and Zn2p_1/2_, respectively. The narrow line widths (2 eV) of these peaks indicate that Zn^2+ ^ions are dominant in the samples as highlighted in Figure 3b. The Mn2p_3/2 _of Zn_0.93_Mn_0.07_O HMSs is observed at 640.8 eV, indicating the existence of Mn^2+ ^ions in microspheres (the metallic Mn and Mn^4+ ^ion should be at 637.7 and 642.4 eV, respectively) [30]. The results previously discussed are consistent with other reports as well [31]. The XPS data of Zn2p and Mn2p further provide evidences for the incorporation of Mn^2+ ^ions into ZnO.

**Figure 4:**
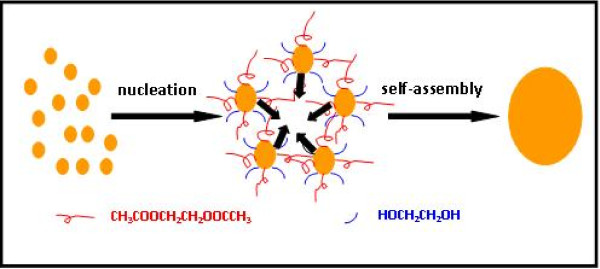
**Illustration of the formation process**.

To obtain a better understanding of the formation of Mn-doped ZnO HMSs, a probable growth mechanism can be proposed. As far as we know, as a wurtzite structure, ZnO belongs to the P63mc space group, and it can be described as a number of alternating planes composed of tetrahedrally coordinated O^2- ^and Zn^2+ ^ions, staked alternately along the *c*-axis. The positively Zn^2+^-terminated (001) polar surfaces of ZnO have the maximum surface energy, while the negatively O^2-^-terminated (00-1) polar surfaces have the minimum surface energy. As a result, the crystal of ZnO nanostructures grows preferentially along the (001) direction, while the growth velocity along other directions is relatively low. Thus, one-dimensional [1-D] structure of ZnO is easily formed. However, why does Mn-doped ZnO in our experiments form globular structures instead of a 1-D structure? We think that EG has a great influence on the morphology of the fabricated Mn-doped ZnO. EG is a polar solvent with a high boiling point (approximately 198°C) and a high permittivity of 37 at 20°C, which is believed to have a high dissolving capacity for polar inorganic materials. Moreover, it is also a strong reducing agent. Here, we propose a two-step self-assembly process for the synthetic Mn-doped ZnO HMSs. As shown in Figure 4, the first route depicts the formation process of Mn-doped ZnO nanoparticles due to the reducing capacity of EG. According to the report, acetaldehyde (CH_3_CHO) is produced by dehydration of EG at a high temperature, where CH_3_CHO can donate a hydrogen atom (H) and can act as a reducing agent as shown in Equation 1 [32]. Consequently, Mn-doped ZnO nanoparticles could nucleate through the reduction of Zn(Ac)_2_/Mn(Ac)_2 _with the newly produced hydrogen atom which is expressed in Equation 2. The second route is the coordination route of EG which directs the formation of Mn-doped ZnO HMSs in view of morphology transformation. A lot of EG molecules are adsorbed on the (001) surface of the ZnO crystals and block the crystal growth along both sides of the *c*-axis. Apart from this, acetic acid could react with EG by esterification (Equation 3), which enlarges the surface modification effect of these small molecules on the surface state [33]. The fresh Mn-doped ZnO crystalline nanoparticles with a large exposed surface are unstable and tend to aggregate to form metastable spheres, driven by the minimum surface tension and the lowest energy for the spherical structure, and finally lead to the formation of globular structures:

(1) $$\text{2HOC}{\text{H}}_{\text{2}}\text{C}{\text{H}}_{\text{2}}\text{OH}\to \text{2C}{\text{H}}_{\text{3}}\text{CHO}\to \text{C}{\text{H}}_{\text{3}}\text{COOC}{\text{H}}_{\text{3}}+\text{ 2H,}$$

(2) $$\text{Zn}{\left(\text{Ac}\right)}_{\text{2}}/\text{Mn}{\left(\text{Ac}\right)}_{\text{2}}+\text{ 2H}\to \text{Z}{\text{n}}_{\text{1}-x}\text{M}{\text{n}}_{x}\text{O }+\text{ C}{\text{H}}_{\text{3}}\text{COOH,}$$

and

(3) $$\text{HO}{\left(\text{C}{\text{H}}_{\text{2}}\right)}_{\text{2}}\text{OH }+\text{ 2C}{\text{H}}_{\text{3}}\text{COOH}\to \text{C}{\text{H}}_{\text{3}}{\left(\text{COOC}{\text{H}}_{\text{2}}\right)}_{\text{2}}\text{C}{\text{H}}_{\text{3}}+\text{ 2}{\text{H}}_{\text{2}}\text{O}\text{.}$$

Based on the mentioned analysis, EG plays at least a triple role. Firstly, it acts as a solvent, providing a medium for the reagents. Secondly, it serves as the reductant which reduces Zn(Ac)_2_/Mn(Ac)_2 _to Mn-doped ZnO crystalline nanoparticles. Finally, it coordinates Mn-doped ZnO nanocrystals to direct the formation of Mn-doped ZnO HMSs.

**Figure 5:**
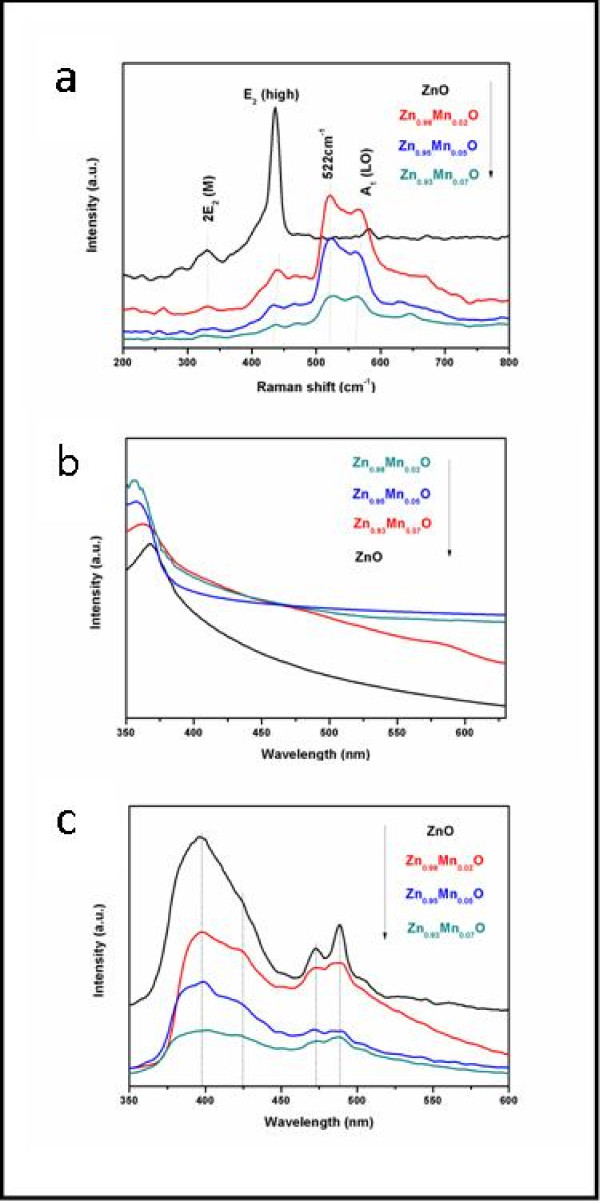
**Raman scattering (a) and UV-vis absorption (b) spectra, and PL spectroscopy (c)**.

Raman spectroscopy is a fast and powerful technique for studying dopant incorporation, induced defects, and disorder in the ZnO host lattice. Raman spectroscopy has also been used to examine crystal quality for various ZnO nanostructures or to find possible secondary oxide phase in magnetic transition ion-doped ZnO. Wurtzite hexagonal-shaped ZnO belongs to the P63mc symmetry group, with two formula units per primitive cell, where all of the atoms are occupying the C_3V _sites. According to the group theory, single crystalline ZnO has eight sets of optical phonon modes at Γ point of the Brillouin zone, classified as A_1 _+ E_1 _+ 2E_2 _modes (Raman active), 2B_1 _modes (Raman silent), and A_1 _+ E_1 _modes (infrared active). Moreover, the A_1 _+ E_1 _modes are polar and split into the transverse optical and longitudinal optical [LO] phonons. E_2 _modes with two modes of low and high frequencies are Raman active only, while the B_1 _modes are silent. Figure 5a shows the Raman spectra of the Zn_1-*x*_Mn*_x_*O HMSs (*x *= 0, 0.02, 0.05, and 0.07). The sharpest and strongest peak at about 437 cm^-1 ^can be assigned to the high frequency branch of the E_2 _mode of ZnO, which is the main Raman mode in the wurtzite crystal structure. It is related to the motion of oxygen atoms and sensitive to internal stress [34,35]. While the peak at about 580 cm^-1 ^can be assigned to A_1_(LO) mode, it is sensitive to changes in the free carrier concentration [36]. Origin of the above mode may be caused by the Zn interstitial. The 331 cm^-1 ^frequency is the second-order vibration mode arising from the E_2_(high)-E_2_(low) multiple scattering process. For all samples, the decreased intensity of the peak at about 437 cm^-1 ^with Mn doping further indicates that the crystallization of the HMSs becomes worse. In addition, the peaks of E_2_(high) and A_1_(LO) mode that shift to lower frequencies with the increase of Mn content indicate that the tensile stress has become increasingly large in the crystal. The tensile stress may come from the increase of defects such as vacancies and interstitials that arose from Mn^2+ ^substituted for Zn^2+ ^in the ZnO matrix. Notably, in comparison with the Raman spectra from undoped ZnO, the anomalous broad band starts to appear from the 500- to 600-cm^-1 ^region, which is named as an indicator of the Mn incorporation into the ZnO matrix [37,38]. A recent report has shown that the anomalous modes in the 500-to 600-cm^-1 ^region have a symmetry, and the Raman scattering forming these modes has a resonance with the Mn^2+^-related optical absorption. The authors have justified the Raman spectral distribution in this region as the Fermi resonance interaction between the overtone 2B and one-phonon A_1_(LO) modes [37].

Figure 5b shows the UV-vis absorption spectra of ZnO with different Mn concentrations. The absorption edges of Zn_1-*x*_Mn*_x_*O (*x *= 0, 0.02, 0.05, and 0.07) are 370, 363, 358, and 355 nm, respectively. The position of the absorption spectra is observed to shift towards the lower wavelength side with increasing Mn-doped concentration in ZnO. This indicates that the bandgap of the ZnO material increases with the doping concentration of the Mn^2+ ^ion. The increase in the bandgap or blueshift can be explained by the Burstein-Moss effect [39]. This is the phenomenon that the Fermi level merges into the conduction band with increasing of the carrier concentration. Thus, the low energy transitions are blocked. The results are in good agreement with the results reported by Sakai and Rekha [40,41].

The optical emission properties of the Zn_1-*x*_Mn*_x_*O HMSs (*x *= 0, 0.02, 0.05, and 0.07) were investigated by PL spectroscopy (Figure 5c) using a 330-nm excitation wavelength of Xe laser at RT. Typically, the following two bands have appeared in the PL spectra: the near-band edge emission in the UV region, which originates due to the recombination of free excitons through an exciton-exciton collision process, and the deep level emission in the visible region, caused by impurities and structural defects of the crystal. Herein, we found that our samples exhibit a UV emission peak at 395 nm and three defect peaks corresponding to blue emissions near 425 and 475 nm and to green emission near 490 nm in the PL spectra. The origin of the peaks at 425 and 475 nm could be ascribed due to the transition occurring from Zn interstitials to the valence band, and the peak of 425 nm may be the result of the singly ionized oxygen vacancy [42]. The increase of the Mn concentration leads to the intensity reduction of both UV and blue emissions, which is mainly due to the increase of defect concentration induced by Mn doping. Besides, the worse crystallization caused by Mn doping is another reason for the rapid decrease in UV emission intensity [43]. This is in agreement with the Raman spectra.

**Figure 6:**
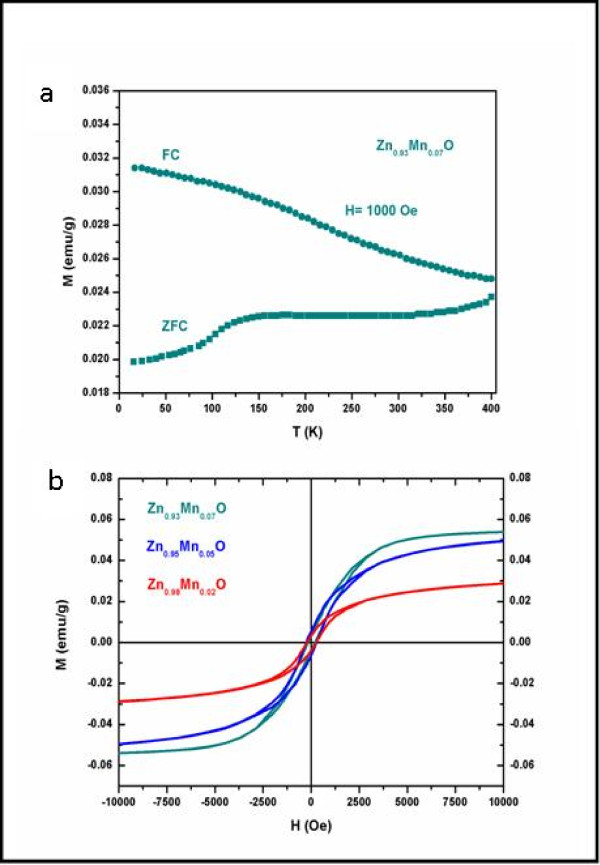
**Magnetization**. The magnetization as a function of temperature for (**a**) FC and ZFC (Zn_0.93_Mn_0.07_O HMSs); RT M-H (**b**) of Zn_1-*x*_Mn*_x_*O HMSs (*x *= 0.02, 0.05, and 0.07).

The magnetization versus temperature properties were characterized using Quantum Design's SQUID magnetometer equipped with a 7-T magnet in the temperature range of 4 to 400 K. Figure 6a shows the magnetization of Zn_0.93_Mn_0.07_O HMSs as a function of temperature obtained at the zero-field-cooled [ZFC] and field-cooled [FC] processes with an applied magnetic field of 1,000 Oe. It is evident that the Zn_0.93_Mn_0.07_O HMSs are ferromagnetic with *T*_C _higher than 400 K due to the clear separation between the FC and ZFC processes. The ZFC-FC magnetization curves clearly indicate that the sample is quite thermally stable without blocking (or superparamagnetic behavior), which is similar to an earlier report on Mn-doped nanoparticles [44]. In addition, magnetic hysteretic loops for the samples with three levels of Mn doping from SQUID measurements are shown in Figure 6b. The magnetization versus magnetic field [M-H] loops for the Zn_1-*x*_Mn*_x_*O HMSs (*x *= 0.02, 0.05, and 0.07) at RT exhibit the coercive field of approximately 280, 255, and 203 Oe, respectively. The coercivity decreases with increasing manganese doping level. The saturation magnetization [Ms] values of the three samples were found to increase with increasing manganese doping. Nevertheless, Ms per manganese still decreases with increasing content of Mn. The value of Ms is 0.0209 μ_B_/Mn for 2% Mn doping and 0.0144 μ_B_/Mn for 5% Mn doping, and it decreases to 0.0111 μ_B_/Mn for 7% Mn doping. Similar phenomena have been observed in other DMS materials [45,46]. Unlike our previous work [47], here, indirect interaction among Mn^2+ ^centers leads to FM, whereas direct interaction among them leads to anti-FM. With increase in Mn doping, the average distance between Mn^2+ ^ions decreases, resulting in the enhancement of the antiferromagnetic contribution [46]. Furthermore, it should be pointed out that these values are much smaller than the theoretical value of 5 μ_B_/Mn for a free Mn^2+ ^ion [47,48]. This can be attributed to the antiferromagnetic superexchange interactions between adjacent Mn^2+ ^ions [48]. We can therefore attribute the RTFM of the Mn-doped ZnO HMSs to the magnetic coupling between Mn atoms [48].

## Conclusion

To summarize, Zn_1-*x*_Mn*_x_*O HMSs (*x *= 0, 0.02, 0.05, and 0.07) with the hexagonal wurtzite structure are synthesized by a simple hydrothermal method. The results of XRD, XPS, and Raman spectrum confirm that Mn^2+ ^ions are successfully incorporated into the ZnO host lattice at the Zn^2+ ^site. The doping of Mn ions suppressed both near-band edge UV emission and defect-related blue emission, which could be mainly caused by the lattice defect increase due to Mn doping into ZnO lattice. In particular, the magnetic measurements reveal that the as-formed Mn-doped ZnO HMSs have above RTFM.

## Competing interests

The authors declare that they have no competing interests.

## Authors' contributions

Y-MH did the synthesis, performed tests on the samples, and wrote the manuscript. S-MZ carried out the magnetic characterization. S-MZ, S-YL, G-YZ, R-JY, and NL modified the manuscript. S-MZ gave the final approval of the version to be published. All the authors read and approved the final manuscript.
